# Application of machine learning to predict transport modes from GPS, accelerometer, and heart rate data

**DOI:** 10.1186/s12942-022-00319-y

**Published:** 2022-11-16

**Authors:** Santosh Giri, Ruben Brondeel, Tarik El Aarbaoui, Basile Chaix

**Affiliations:** 1 INSERM, Nemesis Research Team, Institut Pierre Louis d’Épidémiologie et de Santé Publique, Sorbonne Université, Paris, France; 2 School of Public Health, Ecole des Hautes Études en Santé Publique, Rennes, France; 3 Department of Movement and Sport Sciences, Faculty of Medicine and Health Sciences, Ghent University, Watersportlaan 2, B-9000 Ghent, Belgium

**Keywords:** Transport mode, Prediction models, Global Positioning System, Accelerometer, Heart rate, Machine Learning

## Abstract

**Background:**

There has been an increased focus on active transport, but the measurement of active transport is still difficult and error-prone. Sensor data have been used to predict active transport. While heart rate data have very rarely been considered before, this study used random forests (RF) to predict transport modes using Global Positioning System (GPS), accelerometer, and heart rate data and paid attention to methodological issues related to the prediction strategy and post-processing.

**Methods:**

The RECORD MultiSensor study collected GPS, accelerometer, and heart rate data over seven days from 126 participants living in the Ile-de-France region. RF models were built to predict transport modes for every minute (ground truth information on modes is from a GPS-based mobility survey), splitting observations between a Training dataset and a Test dataset at the participant level instead at the minute level. Moreover, several window sizes were tested for the post-processing moving average of the predicted transport mode.

**Results:**

The minute-level prediction rate of being on trips vs. at a visited location was 90%. Final prediction rates of transport modes ranged from 65% for public transport to 95% for biking. Using minute-level observations from the same participants in the Training and Test sets (as RF spontaneously does) upwardly biases prediction rates. The inclusion of heart rate data improved prediction rates only for biking. A 3 to 5-min bandwidth moving average was optimum for a posteriori homogenization.

**Conclusion:**

Heart rate only very slightly contributed to better predictions for specific transport modes. Moreover, our study shows that Training and Test sets must be carefully defined in RF models and that post-processing with carefully chosen moving average windows can improve predictions.

**Supplementary Information:**

The online version contains supplementary material available at 10.1186/s12942-022-00319-y.

## Background

There has been an increased focus on active transport in Public Health. Studies have shown that individuals who walk, bike, or use public transport perform more physical activity during travel and are more likely to achieve the recommended requirement in daily physical activity [1, 2]. Building on these data and others, policymakers and urban planners are planning transport systems of cities while considering the promotion of exercise and sport and improvement in active transport and public transits infrastructure but also cost-effectiveness, accident prevention and control of traffic speeds, reforms in transport pricings, and health-related exposures (air pollution, noise, etc.) during travel [3, 4].

Accurate data are needed to orientate planning efforts based on evidence. Bohte et al. [5] highlight that memory or recall biases tends to be present in self-reported data on transport activity. Time spent on public transport is usually overestimated, whereas time spent in a car or walking is underestimated. The emergence of new technology and the use of algorithms offer alternatives compared to surveys for collecting data on travel behaviours [6]. Global Positioning System (GPS) data provide information on travel time, speed, and positional characteristics of the travel. Although very accurate, they are prone to signal losses in some areas and are difficult to process [7, 8]. Accelerometer data provide information on how people move, i.e., their bodily movements on three orthogonal axes [9]. The combined use of devices like GPS receivers and accelerometers provides accurate information for predicting travel modes but requires high computational power to process the vast amount of data. The most effective and accurate approach to capture transport modes is likely to combine the processing of GPS data with a GPS-based prompted recall mobility survey where participants are directly interviewed on the basis of GPS data [2, 10, 11]; however, this is a costly strategy, and therefore, it is also essential to develop prediction algorithm-only solutions for the assessment of transport modes, to be able to collect data for broader samples of participants.

GPS-based classification algorithms have been used to detect travel modes, activity places, and trip destinations [12, 13]. Several studies have assessed travel patterns using data from body-worn or vehicular GPS receivers [5, 7, 11, 14–16]. Feng et al. have shown that the combination of GPS and accelerometer data for transport mode prediction at the trip level was more reliable than using GPS data or accelerometer data only [9]. Ellis et al. [17] used data from GPS receivers and accelerometers in a random forest (RF) classification model to classify each minute of observation in five different categories (standing, sitting, walking/running, biking, and riding a vehicle), with ground-truth data obtained from SenseCam cameras. The same authors also compared several machine learning algorithms to predict transport mode and body posture (biking, bus, car, sitting, standing, and walking) in 1-min windows and found that RF models showed the highest prediction rates [18]. Prediction rates similar to Ellis et al. [18] were obtained by Brondeel et al. who used GPS, accelerometer, and Geographic Information System (GIS) data to predict transport modes at the level of trips whose start and end points were a priori known [19]. Kohla et al. used multinomial regression models to predict eight different transport modes at the trip stage level from GPS and accelerometer data [6]. The study by Shafique et al. based on GPS and accelerometer data, personal attributes from participants, and Google Maps information (Distance Matrix API), using RF with Stepwise Feature Inclusion, classified four transport modes (walk, bicycle, car, and train) [20]. Similarly, using sensor data from GPS receivers and accelerometers and utilizing a supervised machine learning method called Support Vector Machine (SVM), Roy et al. predicted five different modes of transport (bicycle, bus, motor vehicle, sky train, and walk) in their study [21].

Most previous studies used either GPS, accelerometer data, or both. There have only been a few attempts to consider heart data (HR) to predict transport modes. Previous studies have been successful in the recognition of various human activities by using accelerometer data complemented by HR data. Ellis et al. in another study than the one mentioned above, were successful in classifying different household and locomotion activities with a 88% accuracy using RF [22]. Although Ellis et al. [22] stated that the inclusion of HR data did not improve the prediction model, Mehrang et al. reported a 7% increase in the prediction of cycling with the inclusion of HR data in the model [23]. The authors also mention the difficulty in accurately differentiating cycling from sedentary behaviour due to the limited motion of the hands while cycling, which can be overcome by the addition of HR data that can help distinguish between activities of different intensities. Finally, comparing various machine learning methods to detect human activities, Balli et al. determined that the RF method was the most successful in predicting eight different human activities in their study, also aided by HR data [24]. The present study, therefore, used heart rate data in addition to GPS and accelerometer data to predict transport modes (rather than activities in a more general way in most of the studies above). Situations where the inclusion of heart rate data may be beneficial are attempts to distinguish between sitting in a car with only minor hand and leg movements while driving vs. standing or moving within a public transport vehicle. Indeed, it is known that changing postures from sitting to standing and vice-versa contributes to even higher elevated HR [25]. Using heart rate data in addition to waist-worn accelerometers may also be beneficial for the identification of cycling.

Moreover, there is room for improvement of prediction algorithms. The present study aimed to demonstrate that, when predicting modes at the minute or trip levels with RF, splitting observations between the Training and Test sets at the minute or trip level upwardly biases prediction rates because data from the same participants are used to train the model and to validate it. This bias is expected to be overcome with participant level split between the Train-Test sets, for example, using the leave-one-out cross-validation method. Moreover, when predicting modes at the minute level, a posteriori processing of predictions is needed to reduce heterogeneity in the minute-level predictions, commonly done with moving averages [18]. We examined whether the bandwidth size used for the moving average influenced the rate of correct predictions, with the assumption that the posterior smoothing of output predictions can reduce the misclassification of transport modes and that both insufficient and excessive smoothing should be avoided.

Overall, accurately predicting transport behaviours and transport modes used from sensor data in an automatic way is a crucial step for increasing the sample size and the quality of studies, which ultimately will be useful to provide urban planners and other policymakers with relevant information on the relationships between transport behaviour and environmental exposures, transport-related physical activity, and health status.

## Methods

### Study participants

**Fig. 1:**
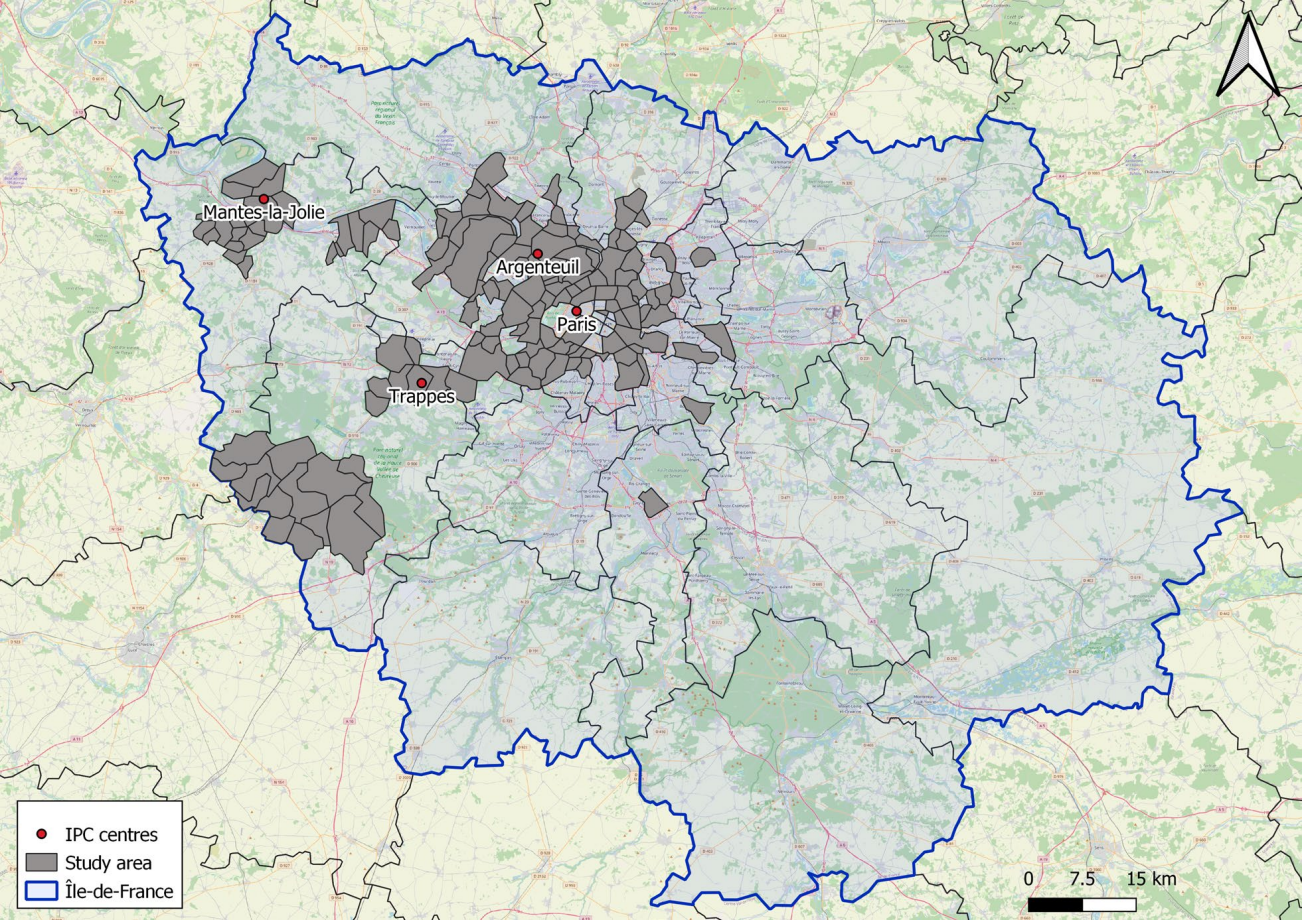
Location of the IPC centres and the study area

The RECORD (Residential Environment and Coronary heart Disease) Cohort Study recruited participants during preventive health check-ups in 2007–2008 at four different sites of the IPC Medical Centre located in Paris, Argenteuil, Trappes, and Mantes-la-Jolie [26]. These participants were invited to the second wave of the study in 2011–2015, and new participants were recruited. The criteria for inclusion in the study were (i) to be 30–79 years year old in 2007–2008, (ii) residing at baseline in one of the 112 municipalities of the Ile-de-France Paris region that had been selected, and (iii) being able to complete study questionnaires [2, 26]. During the second wave of the study, between July 2014 and June 2015, participants were additionally invited to participate in the RECORD MultiSensor Study [10, 27, 28] when sensor devices were available. Figure 1 shows the location of the four IPC centres and the study area. All the participants filled out an informed consent form. The study was approved by the French Data Protection Authority.

Participants wore a GPS receiver (QStarz BT-Q1000XT) [29] and an ActiGraph GT3X + tri-axial accelerometer on the right hip with a dedicated elastic belt for the recruitment day and seven additional days. In addition, they were asked to complete a travel diary to record their visited places over seven days [2, 19]. For heart rate, participants were asked to wear a BioPatch BHM 3 (Zephyr Technology, Annapolis, MD) on the chest for seven days. The BioPatch is an electrocardiography (ECG) sensor with two electrodes [27]. Participants were instructed to remove the GPS receiver, accelerometer, and heart rate monitor only while sleeping and when they were in contact with water and to recharge the GPS receiver and heart rate monitor overnight. Before the data collection was initiated for each participant, synchronization of the sensors was performed. The GPS receiver, accelerometer, and heart rate monitor were all synced with the Internet-based computer time using the dedicated software provided by the manufacturer. Based on these synced timestamps, the variables obtained from the devices were aggregated and merged to create a single-minute level dataset that included all three sensor data from the participants.

One-hundred-twenty-six participants from the “Cardiovascular” arm of the RECORD MultiSensor Study (n = 286) carried a heart rate monitor. This resulted in 334,976-min level observations from 126 participants, for which the sensor data and mode information were available.

### Definition of the outcome to predict from the mobility survey

GPS data collected over seven days were uploaded in a web mapping application called TripBuilder [10] that processed the data with algorithms for the identification of places visited, trips, and trip stages (with the start and end times of all episodes) [11], and transport mode used. Trip information displayed on the computer screen along with the travel diary filled by the participants were used during a prompted recall mobility survey administered via phone calls. Participants confirmed or corrected all information on trips and visited places during the survey. All this information was used to build a timetable containing detailed trip and trip stage information with start and end times of trip stages and corresponding transport mode information for each participant over seven days. This is considered the true information on transport mode in each trip stage to predict.

The outcome to be predicted defined from the GPS-based mobility survey included the following categories: being at a fixed visited location (activity place), walking, biking, using public transport, and using a private motorized vehicle. Thus, our algorithm had to simultaneously predict the fact that participants were travelling rather than at a fixed location and the transport mode when travelling.

### Potential predictors of transport modes

The RF model can handle a large number of predictors, including highly correlated variables. Predictor variables were created from GPS, accelerometer, and heart rate data [27, 30] for every minute of the follow-up of participants. The complete list of predictors is reported in Additional file 1: Table S1.

The GPS device recorded positional data (latitude, longitude, and elevation), speed, and three other measurements: horizontal, vertical, and positional dilution of precision (HDOP, VDOP, and PDOP, respectively), which are the indicators of the quality of the GPS points. Only good quality observations (based on the threshold rule of HDOP < 6, VDOP < 7, and PDOP < 8) were kept for the aggregation of GPS points at the minute level. Standard measures of central tendency (mean and median) and measures of dispersion (standard deviation, minimum, maximum, 10th, and 90th percentiles) were calculated from the GPS positional data, speed, the horizontal, vertical, and positional dilution of precision (HDOP, VDOP, and PDOP respectively), and from the numbers of satellites in view and used for the prediction [19]. Based on the acceleration across the x, y, and z axes recorded by the accelerometer, variables created for each 5-s epoch were (1) the number of steps, (2) the energy expenditure based on the Sasaki and Freedson equation calculated using the activity counts, and participant’s body weight [31], (3) moderate-to-vigorous physical activity (MVPA) [31], and (4) sedentary behaviour [32]. Two series of these variables were created based on raw acceleration data using first the standard filter and second the low-frequency extension filter implemented in ActiLife. The low-frequency extension filter helps detect very low-intensity movement and, consequently, adds information to the prediction process [33].

Heart rate data were collected as time series of intervals between heartbeats, also known as inter-beat (RR) intervals [27]. Heart rate variability parameters were calculated based on the RR intervals, using the “RHRV” R package [34]. All these variables were aggregated at the minute level, as shown in Table 1. Other predictors included time-related variables: time of the day and weekend vs. weekdays. After aggregating the trip level data to the minute level, an average number of 2659.5 data points (Standard Deviation: 1291.1) were available for each participant.

**Table 1:** Overview of the size of the sample used in the study

	GPS	Accelerometer	Heart rate (HR)
**Sample size**			
No. of participants	283	285	127
No. of minute-level observations	1,611,875	2,651,735	566,092

After merging GPS, accelerometer, and HR data			
No. of minute-level observations	336,014		
No. of removed observations	2,316,759		
No. of participants	126		
Final size of the merged dataset	334,976		

### Statistical analysis

Random forest (RF) models [35] were used to predict transport mode at the minute-level from minute-level observations. RF models use an ensemble of decision trees rather than a unique tree and prevent overfitting by using random subsamples of observations for each tree through multiple iterations. For a given N number of trees, N subsamples (train samples) of observations are selected from the dataset. Each subsample is a bootstrap sample from the cases (with replacement) of the same size as the original sample. On average, 63% of observations are included in a particular train subsample, and the remaining 37% are called out-of-bag observations. The train subsample is used to train a decision tree based on a random subset of the explanatory variables, making each tree unique, while the out-of-bag sample is used as a test set for the particular tree. RF classification models use an impurity index called Gini as a decision criterion at each node. Every time a split is made on a node based on a variable, the Gini impurity for the two descendent nodes has to be less than for the previous (parent) node. The outcome that is predicted the largest number of times for a minute-level observation is withheld as the final prediction for this observation. We examined the evolution of the error rate as the number of trees was incrementally increased. Based on the drop in the error rate, models were built with 100 trees, using default parameters.

An issue with the standard application of RF used in previous research of this kind is that the split of minute-level observations between the train and test sets for each particular tree ignores the fact that the same participants provide minute-level observations to both the train and test sets. It obviously favours overfitting to specific participants and spuriously increases the prediction rate in the test set. As a remedy, we split our sample at the participant level, with data from 125 participants in a Training set and the remaining one in a Test set, usually referred to as the leave-one-out cross-validation method. We then ran the RF model within the Training set (of 125 participants), with train and test sets repeatedly defined as usual in the default RF approach in this Training set, and reported the out-of-bag prediction rate, which we think is biased (due to the fact that that the same participants provide observations to train and test sets). We then tested the resulting model in the Test set (containing one unique separate participant), which should provide a correct prediction rate. We repeated the split 126 times, performing leave-one-out cross-validation. Barshan et al. discussed the use of three different cross-validation methods in their study [36]. They reported that the results from leave-one-out cross-validation method was the most meaningful when data from different subjects are partitioned in the training and test sets, such as in our case. Separate models were built with and without heart rate data in each iteration.

**Fig. 2:**
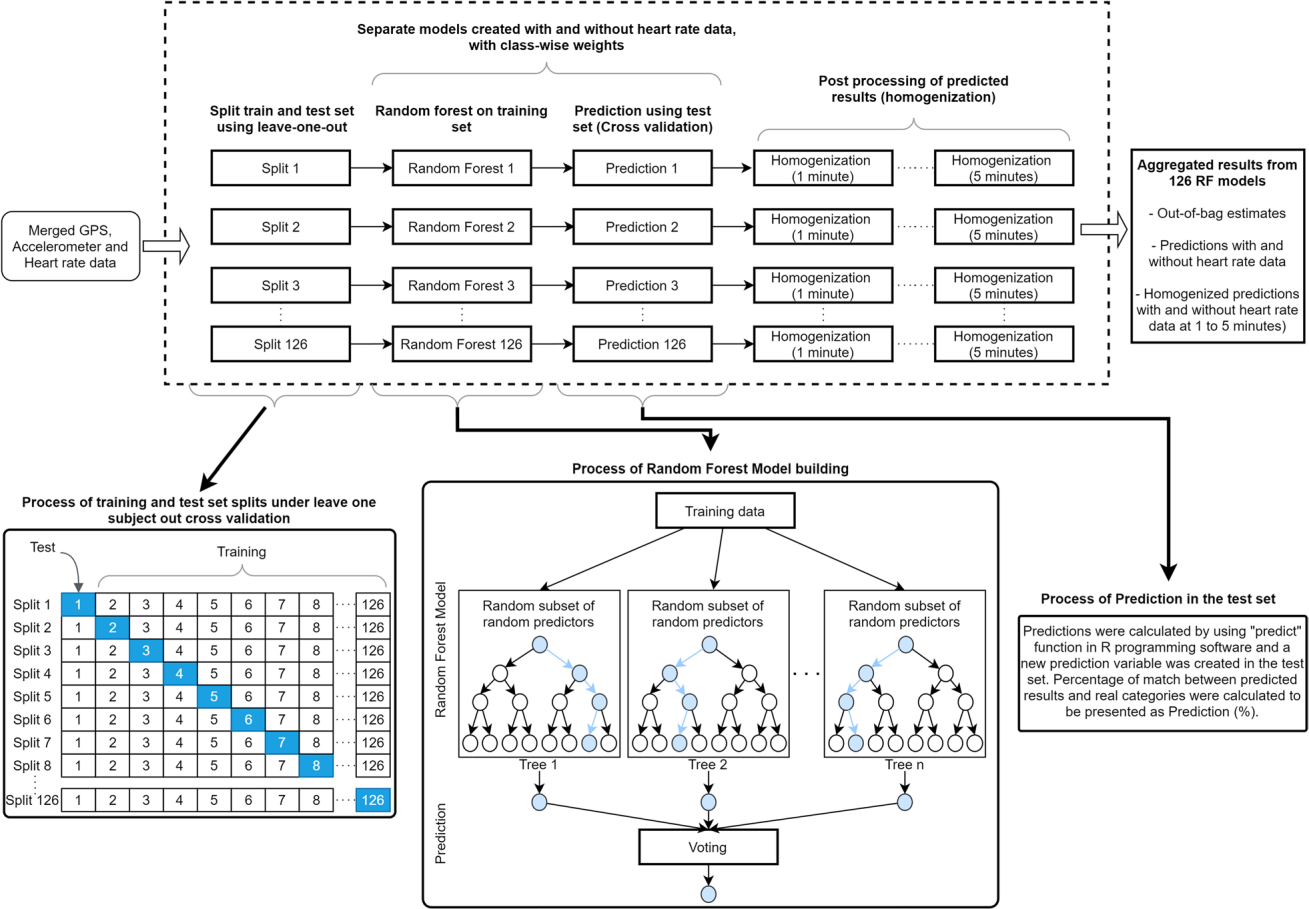
Flow diagram of the analysis process

Regarding the rates of successful prediction by categories of the outcome (i.e., for specific transport modes), it is well known that categories with higher numbers of observations have better prediction rates than categories with fewer observations (RF tends to favour the majority classes). Class-wise weights were also applied to the model to estimate correct prediction rates by categories to fix the issue [37]. The entire process of data analysis is presented in a flow diagram in Fig. 2.

R version 4.1.3 and the “randomForest” package [38, 39] were used. Due to the vast amount of data and multiple iterations for Random Forest, running the prediction models was computationally challenging. Thus, we distributed the analysis between two different computers with multiple windows of R running simultaneously.

### A posteriori homogenization of predictions

As expected, a closer look at the minute-level predictions in the Test set indicated sporadic misclassifications of the predicted transport mode within a trip. A simple moving average output filter was used for the homogenization of misclassified transport modes to improve the prediction rates. The filter processes each minute in sequential order and outputs for the central minute of interest, the mode predicted the highest number of times in the surrounding windows. As a sensitivity analysis, we considered different bandwidths surrounding the minute of interest with a window of 1 to 5 min before and after. For a 1-min bandwidth, if the preceding and succeeding minutes had a similar mode B, then the discordant mode A of the minute under consideration was changed from A to B.

## Results

### Descriptive statistics

Our sample of 126 participants comprised 39% of women, and the mean age was 50.7 (min: 34, max: 77). Seventeen percent of participants did not complete high school, while 52% had a degree corresponding to 3 years or more after high school.

Over the seven days of follow-up, participants performed a median of 5.1 trips per day (10th and 90th percentiles: 2.9, 7.8), corresponding to a median of 8.7 trip stages per day (10th and 90th percentiles: 4.7, 12.6). Participants spent a median of 6.9 percent of their follow-up time on trips (10th and 90th percentiles: 3.6, 11.0), corresponding to a duration of 100.00 min per day (10th and 90th percentiles: 51.4, 158.0).

### Participant-level vs. minute-level split of observations

As shown in Table 2, the overall naïve out-of-bag prediction rate (obtained by splitting observations of the Training sets at the minute level) was similar or comparable to the overall prediction rate derived from the Test sets, and the same applied to the prediction rate for being at a visited place. However, for the overall prediction rate for transport modes and each of the prediction rates for the specific transport modes, as expected, the naïve out-of-bag prediction rates (based on observations from the same participants that were used to grow the forests) were higher than the prediction rates derived from participant-distinct Test sets.

**Table 2:** Comparison of Out-of-bag prediction rates (%) from Training sets and Prediction rates from Test sets

	Naïve out-of-bag prediction rates in the training sets		Prediction rates^a^ in the test sets	
	Before correction	After correction	Before correction	After correction
Overall	94 (94–94)	78 (78–78)	94 (81–98)	79 (62–88)
Overall transport	70 (70–70)	77 (76–77)	64 (33–88)	74 (41–89)
Activity place	98 (98–98)	78 (78–79)	98 (95–100)	79 (61–89)
Bike	67 (66–68)	88 (88–89)	65 (40–82)	90 (77–100)
Private motorized	84 (84–85)	72 (72–73)	85 (16–96)	69 (1–89)
Public transport	46 (44–47)	75 (74–75)	22 (0–68)	62 (0–91)
Walking	63 (62–63)	81 (81–82)	61 (5–88)	80 (23–95)

^a^ Prediction rates presented as median prediction rates from 126 RF models with 2.5th and 97.5th percentiles in the parentheses. Prediction rates by transport modes are shown before and correction for category size; model without heart rate data

### Contribution of HR

As shown in Table 3, adding HR predictors to RF models already comprising GPS and accelerometer predictors did not improve the overall prediction rate. Regarding mode-specific prediction rates, while the final prediction rate for biking slightly increased (by 2 percentage points), no changes in prediction rates were observed for other transport modes.

**Table 3:** Prediction rates of transport modes in the Test sets: models with and without heart rate data

	Prediction rates^a^ (%)	
	Without heart rate data	With heart rate data
Overall	79 (62–88)	79 (57–88)
Overall transport	74 (41–89)	74 (41–89)
Activity place	79 (61–89)	80 (55–89)
Bike	90 (77–100)	92 (76–100)
Private motorized	69 (1–89)	69 (0–87)
Public transport	62 (0–91)	62 (0–90)
Walking	80 (23–95)	80 (28–95)

^a^Prediction rates presented as median prediction rates from 126 iteration of RF models with 2.5th and 97.5th percentiles in the parentheses. The overall prediction rate is from a model that is unweighted by category size, while the mode-specific prediction rates are from a corrected model applying a weight. Correction for category size entails modifying the cut-offs for prediction to the observed proportions of the categories, at the forest prediction step (when aggregating information from all trees). For the same tree predictions, a higher “proportion of votes” is reached for rarer categories in the weighted vs. unweighted model

### A posteriori homogenization

As shown in Table 4, applying a moving average homogenization systematically improved the final prediction rates obtained in our Test sets. Improvements ranged from 2 percentage points for walking to 10 percentage points for using a private motorized vehicle. Depending on the outcome, the curve of improvement of prediction rates peaked when a 3-, or sometimes 4-, or even 5-min bandwidth moving average was used and then either plateaued or decreased when enlarging the bandwidth further.

**Table 4:** Prediction rates of transport modes in the Test sets before and after a posteriori homogenization

	Prediction rates^a^ (%)					
	Before homogenization	1-min bandwidth	2-min bandwidth	3-min bandwidth	4-min bandwidth	5-min bandwidth
Overall	79 (57–88)	86 (68–93)	88 (66–94)	89 (67–95)	90 (67–95)	90 (67–95)
Overall transport	74 (41–89)	76 (39–91)	79 (42–92)	80 (42–94)	78 (40–95)	77 (35–96)
Activity place	80 (55–89)	89 (68–95)	90 (68–96)	91 (69–97)	91 (71–97)	92 (71–97)
Bike	92 (76–100)	93 (77–100)	95 (79–100)	95 (80–100)	95 (78–100)	95 (77–100)
Private motorized	69 (0–87)	73 (0–92)	77 (0–96)	78 (0–96)	79 (0–97)	79 (15–100)
Public transport	62 (0–90)	63 (0–95)	63 (0–95)	65 (0–100)	65 (0–100)	66 (0–100)
Walking	80 (28–95)	81 (29–97)	81 (23–97)	82 (22–97)	81 (21–98)	78 (17–99)

^a^Prediction rates presented as median prediction rates from 126 RF models with 2.5th and 97.5th percentiles in the parentheses. The overall prediction rate is from a model (with heart rate data) that is unweighted by category size, while the mode-specific prediction rates are from a corrected model (with heart rate data) applying a weight. Correction for category size entails modifying the cut-offs for prediction to the observed proportions of the categories, at the forest prediction step (when aggregating information from all trees). For the same tree predictions, a higher “proportion of votes” is reached for rarer categories in the weighted vs. unweighted model

### Final predictions

Our final report of findings considers predictions established in our Test sets (separate participant) from RF models including GPS, accelerometer, and heart rate predictors, after applying a 3-min bandwidth moving average. Moreover, class-wise weights are applied to prevent outcomes with a lower prevalence from being penalized (as shown in Table 2, this correction tended to increase the prediction rates of biking, using public transport, and walking, while it decreased the prediction rate for being at a visited place and using a personal motorized vehicle).

As shown in Table 4 (sixth column), the overall prediction rate was 90%. However, such a prediction rate is markedly increased by the correct prediction of being at visited locations (e.g., at home or work), which accounts for a significant proportion of the time. Therefore, we also calculated a prediction rate that only pertains to the prediction of transport modes. This overall prediction rate of transport modes was 78%. The model and post-processing achieved a prediction rate of 95% for biking and 81% for walking, while the rate of correct prediction was only 65% for public transport. Using a private motorized vehicle achieved a better prediction rate without correcting for category-specific weights.

## Discussion

The diversity in the availability of methodologies (machine learning techniques vs. deep learning approaches), data sources (public vs. tailored for study), sensors and instruments, and approaches in pre-post-processing opens a multitude of avenues in transport mode detection. Our study used RF models to predict transport modes at the minute level. We examined the added impact of heart rate in the prediction, assessed the impact of splitting observations at the participant level rather than at the observation level during the estimation procedure, and we investigated the influence of bandwidth size during the post-processing moving average on the final prediction rate. Various recent studies have approached transport mode detection with different methods like classical machine learning techniques (RF, SVM) [40, 41], Convolutional Neural Network (CNN) [42], Long Short-Term Memory (LSTM) [43], Temporal Convolutional Network (TCN) [44], Multilayer Perceptron (MLP) [45]. Some have used multiple algorithms as an evaluation of their chosen method in their study [40–42, 44–46]. For example, Alotaibi, in their article, presents an ensemble method that utilized a combination of three different machine learning algorithms for classification or model learning [45]. They used two additional algorithms stacked with the ensemble fed into a neural network architecture named multilayer perceptron (MLP), in order to predict five different transport modes. While the use of this multilayer algorithm for model building and prediction outperformed the predictions of twelve other independent machine learning algorithms in their study, the use of smartphone motion sensors to create a 5-s window size for their dataset vastly differs from ours where we use minute level observations of GPS, accelerometer and heart rate.

Similar use of smartphone sensors at 4-s window size was presented by Mantellos et al. where they proposed a smartphone application to automatically predict the transport modes only using motion-based sensors and a rule-based algorithm known as PART [47]. Their study, focusing on the development of a smartphone application to create environmental awareness to enable people to follow a sustainable way of life, also gave significant attention to the comparison of different algorithms to select a method that was simple to use for the automatic recognition of transport modes using smartphones. The study also reported a difficulty similar to ours to differentiate between being in a car vs. in a bus. We attempted to overcome this problem by using heart rate data, which Mantellos et al. [47] report as a potential improvement for their future work by following a hierarchical classification approach like RF, and for classifying different motorized and non-motorized transportation modes. Some recent studies have also used shorter window sizes (ranging from 5 to 8.7 s) [43, 44, 46] while others used trips [40–42] for the transport mode detection in their study.

Most of the recent studies in our literature review have used accelerometer, gyroscope, and magnetometer data derived from smartphones [42–47], with one study using GPS only [40], and one using the GPS, accelerometer, and heart rate data from a smartphone and smartwatch as in our study [41]. Although smartphones make it easy to collect mobility data, sensor information may vary across different smartphone devices compared to bespoke sensor devices (like the ones used in our study) whose primary function is to collect sensor data. For reliable accelerometer measures, the smartphones would have to be well attached to a fixed place on the body (for example, on the hips) and basically not be used as a smartphone. Moreau et al. and Wang et al. used a real-world dataset for their study, which contains multi-modal data collected using a body-worn camera and multiple smartphones fixed at typical body locations [42, 44]. Using this dataset, Moreau et al. [42] predicted six transport modes with 98% precision using CNN, while Wang et al. [44] predicted eight transport modes with a precision of 87% using TCN. Deep learning methods require more pre-processing, have complex training processes, and are computationally expensive compared to RF. According to Hasan et al. RF was the most accurate in identifying different transport modes when comparing Extreme Gradient Boosting, RF, SVM, and ANN in their study, where they used GPS, accelerometer, and heart rate data at trip level [41]. They employ a similar web-based application for trip generation as we used to identify trips and trip stages in the GPS-based mobility survey. One difference though is that we further disaggregated the trips into minutes of observation, because we did not want to assume that the trips’ start and end times were a priori known. To the best of our knowledge, no recent study has attempted to predict the use of transport modes at the minute level while accounting for GPS, accelerometer, and heart rate with post-processing done via homogenization.

### Main results

#### Participant-level vs. observation-level split of observations

In the model without heart rate, the naïve out-of-bag prediction rate for being in transport in the Training sets was 77%. As expected, the overall prediction rate for being in transport was lower (74%) in our Test sets. In the Training sets, RF splits minute-level observations from the same participants between the train and test samples. Thus, the naïve out-of-bag prediction rate in the Training set is upwardly biased because it is based on observations from the same participants that were used to train the model. The overall prediction rate from our Test sets provides more accurate information on the model’s performance as it is derived from a different participant than those used to grow the model, which is definitely what we aim for with our prediction efforts. Because the participant-level split between the Training set and Test set was repeated 126 times, the unbiased prediction rate derived from the Test sets takes into account information from all participants.

Brondeel and Chaix achieved a prediction rate of 90% using GPS, accelerometer, and GIS data in an RF model at the trip level [19]. However, because observations from the same participants were used to grow the model and validate it, we expect our reported prediction rates to be upwardly biased. Similarly, using a Bayesian Belief Model, Feng et al. obtained a prediction rate of approximately 90% at the trip level using GPS, accelerometer, and survey data [9]. It is indicated without further details that 65% of observations were assigned to the calibration set while 35% were used as a validation set. If the same participants contributed observations to both the calibration and validation sets, the reported prediction rate should be seen as overestimating the correct prediction rate when applied to a new set of participants.

Ellis et al. used a combination of strategies to predict a mix of body posture and transport mode with their RF model based on GPS and accelerometer data [17]. Some of their strategies were unbiased, such as when models were grown and tested in different samples and when a “leave-one-day-out cross-validation” was used (each time, data from one day was used for testing). Shafique et al. used a mix of data (GPS, accelerometer, personal attributes, and Google Maps information) to reach an impressive 99.6% correct prediction rate using Random Forest with Stepwise Feature Inclusion [20]. Their prediction accuracies ranged from 95.44% to 99.84% for four transport modes (walk, bicycle, car, and train). The authors adopted a more complicated moving average filter employed during the pre-processing to account for the variability in the accelerometer data. The near-perfect accuracy in their study can possibly be attributed to the fact that the train-test split was done at the outcome level (similar to the observation level), where 70% of the data from each transport mode was randomly included in the training set and the rest in the test set, with the same participants providing data to both sets.

Other studies by Gong et al. [48] and Chen et al. [49] have yielded prediction rates of 82.6% and 79.1% at the trip stage level, respectively. Because they used GIS rule-based algorithms, the methodological issue related to RF that we raise does not apply to their work.

#### Heart rate in addition to GPS and accelerometer data

Previous studies have used a combination of GPS, accelerometer, and heart rate data for the prediction of other outcomes such as energy expenditure and physical activity [50–53], but to the best of our knowledge, only very rarely for the prediction of transport modes e.g., for the prediction of cycling or for identification of the most significant variables in transport modes prediction [41]. Our a priori hypothesis that heart rate data would substantially contribute to a better distinction between motorized transport modes (especially between driving a car and using public transport) was not confirmed overall. Probably heart rate provided information strongly correlated to the one provided by accelerometers. Given that accelerometer measurement is easier to implement than heart rate measurement, we did not examine whether accelerometer variables added to prediction accuracy based on GPS and heart rate data. It should be emphasized, however, that heart rate seemed to improve the prediction for biking which is easily understandable as waist-worn accelerometers are unable to adequately capture biking physical activity.

#### Predictive contribution of variables

**Fig. 3:**
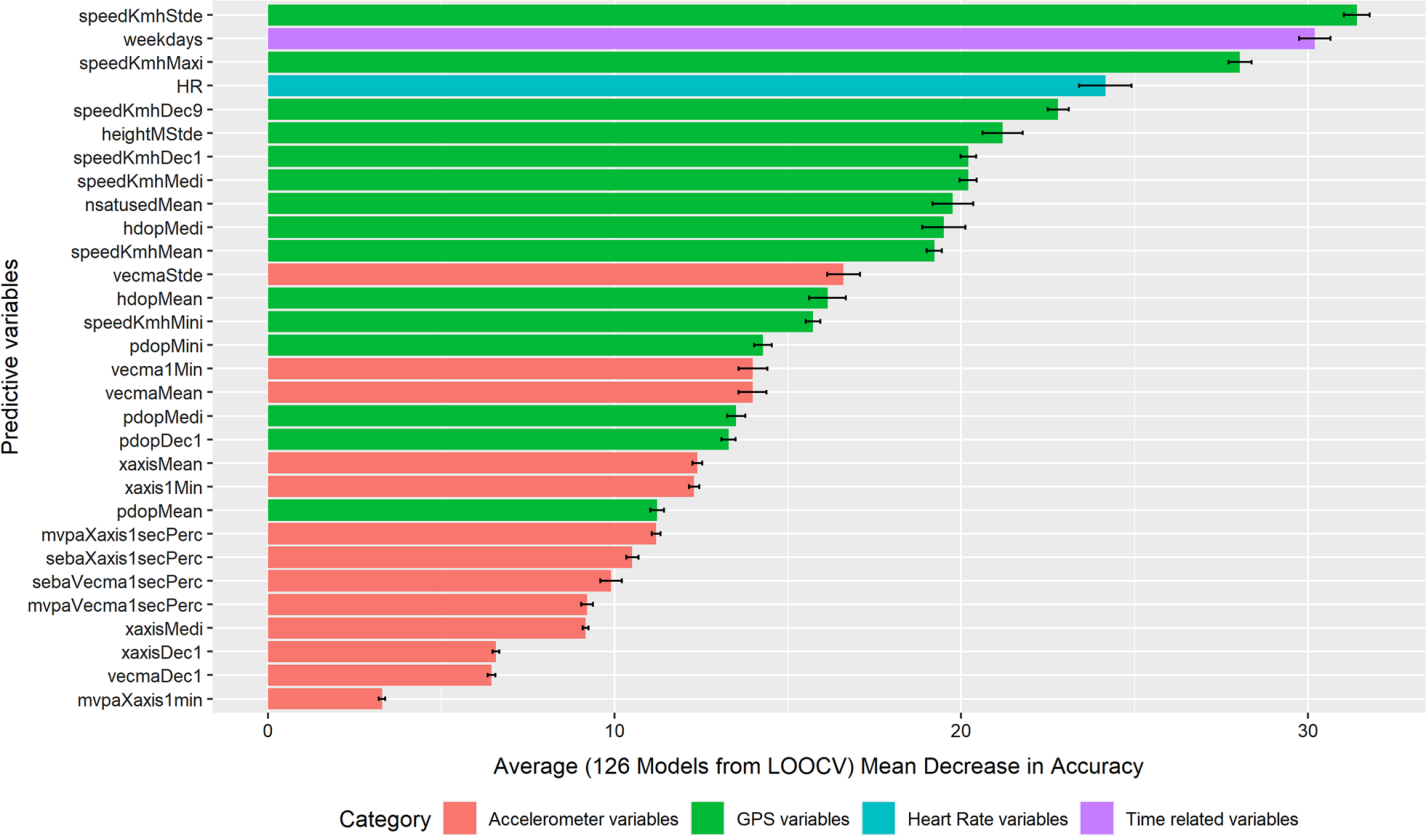
Variable importance plot: Mean decrease in accuracy

**Fig. 4:**
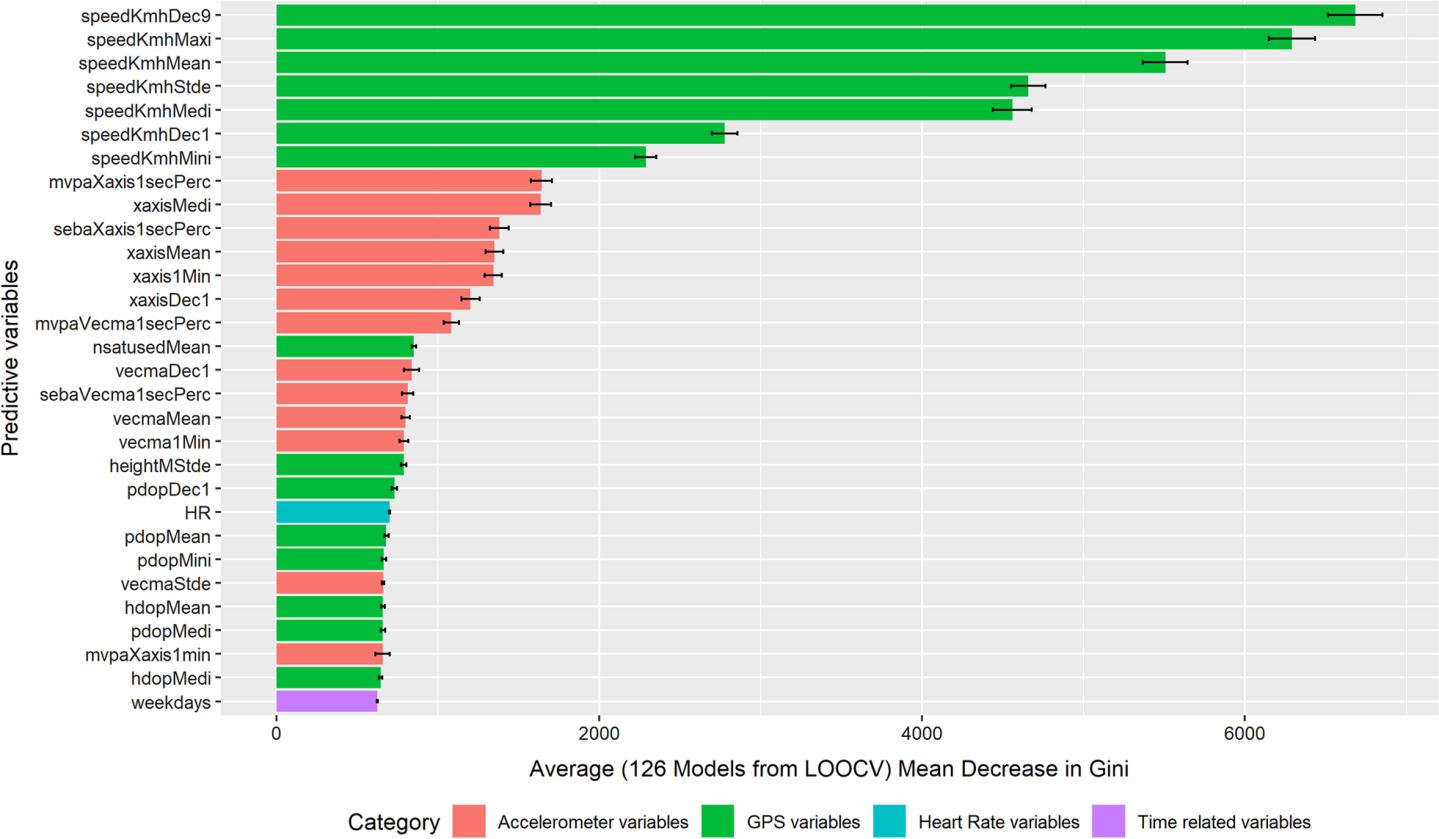
Variable importance plot: Mean decrease in Gini

Predictors that were tested included variables from the accelerometer (55 variables), the GPS receiver (51 variables), and the heart rate monitor (12 variables). The variable importance plots (for models including heart rate data) give a glimpse of which variables contributed most in terms of the overall accuracy of the prediction and related performance of the model (mean decrease in accuracy) as illustrated in Fig. 3, and in terms of purity of the final subgroups in the tree through splits with this variable (mean decrease in Gini) as depicted in Fig. 4.

Half of the predictors out of the top 30 predictive variables contributing to the model’s accuracy belonged to GPS, followed by 13 accelerometer variables, one heart rate, and one time-related variable. It should be noted that none of the accelerometer variables made it to the top 10 contributing predictors. The standard deviation of speed (from GPS data) contributed most in terms of the predictive accuracy of the models (mean decrease in accuracy). The information on weekday vs. weekend was ranked second, followed by maximum speed, heart rate, and then by other speed and GPS indicators. The mean number of satellites used was ranked in the ninth position. While the importance of weekday/weekend (ranked second) was surprising, the importance of speed was expected since speed, and also the variance in speed, differ between transport modes, even between motorized transport modes (for example, public transport vehicles may have a more constant speed and more regular stops, compared to private motorized vehicles). It is interesting to see how heart rate contributed to the predictive accuracy of the entire model even if it was not found to improve the prediction rate in addition to other variables, which is likely due to substitutions among variables. Variables related to the number of satellites in view are likely relevant for predicting public transport, which is often underground in the Paris area. It was assumed that accelerometer data measuring body acceleration would be important, but the model reported otherwise.

In contrast, Ellis et al. and Brondeel et al. found that accelerometer variables contributed more in terms of predictive power than the GPS data [18, 19]. This is difficult to explain, given that the latter study was based on data collected by our team with comparable methods for another sample over similar territory.

#### A posteriori homogenization of predictions

Our a posteriori homogenization systematically improved the reported prediction rates as we expected. Ellis et al. reported significant improvements in their final prediction rates after using the moving average filter [18]. Similarly, Prelipcean et al. observed higher accuracy growth using their “Explicit-consensus methods” compared to other performance metrics used in their study [54]. This method also used a voting principle on each point of the trip segment but instead took into account the whole trip segment than a window of points before and after the point in question, which strategy is difficult to apply when the trip start and end is not known beforehand as in our case.

In our study, using the moving average filter, the successful prediction rate of transport modes (overall) was increased by 6 percentage points. In comparison, the prediction of public transport use was improved by 4 percentage points and that of using a private motorized vehicle by 10 percentage points. Thus, our study demonstrates that the posteriori homogenization reasonably improved the final prediction rates, although not substantially, and that it is a useful step in a prediction process.

There was evidence that excessively large homogenization windows tended to obscure the prediction of walking episodes, which are typically shorter than those with other modes. Thus, our work suggests that investigators need to pay close attention to the size of the homogenization window and that a unique window size may not identically apply to all transport modes.

#### Overall and mode-specific prediction rates

Although the overall prediction rate (in our Test sets, after applying a posteriori homogenization) seemed high (90%), it was greatly influenced by the extended stays at places visited that are relatively easy to predict (91%). We addressed this issue in two ways, first by deriving an overall prediction rate for transport modes (excluding stays at visited places), which was 80% at its peak, and second by calculating mode-specific prediction rates. For the latter approach, we applied class-wise weights based on the observed proportion of the modes among all trips, which prevents rare modes from having their prediction rate penalized due to their low prevalence [19, 37]. Our final prediction rate of transport modes (80%) suggests that there is room for improvement for our model of prediction of being in trips rather than a visited place and of transport modes. Biking achieved a higher prediction rate than the other modes. Particular efforts are needed in the future for predicting public transport use, for example, taking into account the location of public transport stations to aid the prediction.

### Strengths and limitations

The main strength of this study is that it relies on a large sample of accurately identified trips using GPS tracking and a GPS-based mobility survey. Our sample includes a large number of trip configurations from participants in free-living conditions, along with a large number of different types of personal motorized vehicles and public transport vehicles. Thus, it is logical to expect a lower rate of correct prediction in our study than in others with less variability in trip conditions. Still, the relatively low final prediction rate is the major limitation of this work, which will have to be improved in the future. Moreover, the region of Paris has a specific transport system with a densely connected public transport network with highly walkable areas. Our particular prediction model is likely not generalizable to contexts with different transport and urban systems; however, our data collection and data processing methodology is the second strength is that our study is one of the first to include heart rate data for the prediction of transport mode. However, the contribution of heart rate data to improve the prediction of private and public transport modes, as hypothesized, was not verified in our analyses, and the inclusion of heart rate data only very slightly improved the prediction of biking. A third related strength is the large set of potentially relevant predictors generated for our modelling from GPS, accelerometer, and heart rate data.

The fourth strength is that we developed an algorithm for minute-level prediction, which assesses both being at a visited place and being on trips. Thus, contrary to our previous work [19], the present algorithm is a standalone algorithm that does not require a pre-identification of trips. In the present work, we tested an alternative two-step approach where we first used an algorithm based on GPS speed to identify trip stages, and in a second step, attempted to predict transport mode at the trip stage level and then compared the predictions to the ground-truth from our GPS-based mobility survey. However, this approach yielded abysmal prediction rates due to the combination of uncertainty in the first step (identifying trips) and the second step (identifying modes). The fifth strength of the work is related to the methodological developments implemented, including improving over the straightforward split of observations in RF ignoring the nesting of observations within individuals and investigating the impact of the window size for the a posteriori homogenization of predictions.

Limitations to overcome in the future include the restricted age range of our sample population. Future considerations should be given to expanding the selection of participants to include a large age range (preferably 18 years and above). Also, the inclusion of participants’ sociodemographic characteristics should be considered to determine whether personal information can contribute to prediction accuracy. Finally, the application of our method to mobility data from various and differing urban settings would provide the level of variability our algorithm needs to improve the generalizability of our findings.

### Conclusions

Our study shows that it is feasible to use sensor-based prediction models of transport modes. Our work suggests that GPS and accelerometer data provide relevant information for the prediction and that heart rate adds minor information only for specific transport mode. Finally, our work demonstrates that a two-phase approach, including RF prediction and a posteriori homogenization, improves over RF prediction only.

## Supplementary Information

**Additional file 1:** **Table S1.** List of all the predictors generated from GPS, accelerometer, and heart data used in the Random Forest prediction.

## Data Availability

The datasets generated and/or analysed during the current study are not publicly available due to the sensitivity of data (GPS receiver data providing the location of participants) but are available from the research team in the context of scientific collaborations.

## Abbreviations

ECG: Electrocardiography
GIS: Geographic Information System
GPS: Global positioning system
HDOP: Horizontal Dilution Of Precision
MVPA: Moderate-to-vigorous physical activity
PDOP: Positional dilution of precision
RECORD: Residential environment and coronary heart disease
RF: Random forest
VDOP: Vertical dilution of precision
