# Supplementary material for: Application of machine learning to predict transport modes from GPS, accelerometer, and heart rate data
Source: Int J Health Geogr. 2022 Nov 16;21:19. doi: 10.1186/s12942-022-00319-y (PMC9667683; doi:10.1186/s12942-022-00319-y)
Supplement: Supplementary file 1 — Additional file 1: Table S1. List of all the predictors generated from GPS, accelerometer, and heart data used in the Random Forest prediction. [file 12942_2022_319_MOESM1_ESM.docx]

Additional file 1: List of variables used for prediction modeling

Table S1. List of all the predictors generated from GPS, accelerometer, and heart data used in the Random Forest prediction

| **Variables** | | **Definition** |
| --- | --- | --- |
| pid | | Participant ID (not included in the model) |
| idx.time | | Unique ID for the minute level (not included in the model) |
| **Time related variables** | | |
| time | | Time interval of each minute (not included in the model) |
| weekdays | | Weekdays |
| weekend | | Weekends |
| timeOfDay | | Time of day (day or evening) |
| **Accelerometer variables (all variables are included both with the standard filter and the low frequency extension filter)** | | |
| inclinometerLyingPerc | | Percentage of epoch lying |
| inclinometerOffPerc | | Percentage of epoch the accelerometer was off |
| inclinometerSittingPerc | | Percentage of epoch sitting |
| inclinometerStandingPerc | | Percentage of epoch standing |
| lipaVecma1min | | Vector magnitude measure of light physical activity at 1 minute |
| lipaVecma1minPerc | | Percentage of Vector magnitude measure of light physical activity at 1 minute |
| lipaVecma1secPerc | | Vector magnitude measure of light physical activity at 1 second |
| lipaXaxis1min | | X-axis measure of light physical activity at 1 minute |
| lipaXaxis1minPerc | | Percentage of X-axis measure of light physical activity at 1 minute |
| lipaXaxis1secPerc | | X-axis measure of light physical activity at 1 second |
| mvpaVecma1min | | Vector magnitude measure of MVPA at 1 minute |
| mvpaVecma1minPerc | | Percentage of Vector magnitude measure of MVPA at 1 minute |
| mvpaVecma1secPerc | | Vector magnitude measure of MVPA at 1 second |
| mvpaXaxis1min | | X-axis measure of MVPA at 1 minute |
| mvpaXaxis1minPerc | | Percentage of X-axis measure of MVPA at 1 minute |
| mvpaXaxis1secPerc | | X-axis measure of MVPA at 1 second |
| sebaVecma1min | | Vector magnitude measure of sedentary behavior at 1 minute |
| sebaVecma1minPerc | | Percentage of Vector magnitude measure of sedentary behavior at 1 minute |
| sebaVecma1secPerc | | Vector magnitude measure of sedentary behavior at 1 second |
| sebaXaxis1min | | X-axis measure of sedentary behavior at 1 minute |
| sebaXaxis1minPerc | | Percentage of X-axis measure of sedentary behavior at 1 minute |
| sebaXaxis1secPerc | | X-axis measure of sedentary behavior at 1 second |
| vecma1Min | | Total vector magnitude per epoch (1 minute) |
| vecmaDec1 | | 10th percentile vector magnitude per epoch |
| vecmaDec9 | | 90th percentile vector magnitude per epoch |
| vecmaMaxi | | Max. vector magnitude per epoch |
| vecmaMean | | Mean vector magnitude per epoch |
| vecmaMedi | | 90th quantile vector magnitude per epoch |
| vecmaMini | | Min. vector magnitude per epoch |
| vecmaStde | | Std. dev of vector magnitude per epoch |
| wearing | | If the accelerometer was worn for each minute |
| xaxis1Min | | Total acceleration on X-axis per epoch (1 minute) |
| xaxisDec1 | | 10th percentile acceleration on X-axis per epoch |
| xaxisDec9 | | 90th percentile acceleration on X-axis per epoch |
| xaxisMaxi | | Max. acceleration on X-axis per epoch |
| xaxisMean | | Mean acceleration on X-axis per epoch |
| xaxisMedi | | 90th quantile acceleration on X-axis per epoch |
| xaxisMini | | Min. acceleration on X-axis per epoch |
| xaxisStde | | Std. dev of acceleration on X-axis per epoch |
| yaxis1Min | | Total acceleration on Y-axis per epoch (1 minute) |
| yaxisDec1 | | 10th percentile acceleration on Y-axis per epoch |
| yaxisDec9 | | 90th percentile acceleration on Y-axis per epoch |
| yaxisMaxi | | Max. acceleration on Y-axis per epoch |
| yaxisMean | | Mean acceleration on Y-axis per epoch |
| yaxisMedi | | 90th quantile acceleration on Y-axis per epoch |
| yaxisMini | | Min. acceleration on Y-axis per epoch |
| yaxisStde | | Std. dev of acceleration on Y-axis per epoch |
| zaxis1Min | | Total acceleration on Z-axis per epoch (1 minute) |
| zaxisDec1 | | 10th percentile acceleration on Z-axis per epoch |
| zaxisDec9 | | 90th percentile acceleration on Z-axis per epoch |
| zaxisMaxi | | Max. acceleration on Z-axis per epoch |
| zaxisMean | | Mean acceleration on Z-axis per epoch |
| zaxisMedi | | 90th quantile acceleration on Z-axis per epoch |
| zaxisMini | | Min. acceleration on Z-axis per epoch |
| zaxisStde | | Std. dev of acceleration on Z-axis per epoch |
| **GPS variables** | | |
| heightMMean | | Mean elevation |
| speedKmhMean | | Mean speed |
| pdopMean | | Mean positional dilution of precision |
| hdopMean | | Mean horizontal dilution of precision |
| vdopMean | | Mean vertical dilution of precision |
| nsatusedMean | | Mean number of satellites used |
| nsatviewMean | | Mean number of satellites in view |
| heightMStde | | Standard deviation of elevation |
| speedKmhStde | | Standard deviation of speed |
| pdopStde | | Standard deviation of positional dilution of precision |
| hdopStde | | Standard deviation of horizontal dilution of precision |
| vdopStde | | Standard deviation of vertical dilution of precision |
| nsatusedStde | | Standard deviation of number of satellites used |
| nsatviewStde | | Standard deviation of number of satellites in view |
| heightMMedi | | Median of elevation |
| speedKmhMedi | | Median of speed |
| pdopMedi | | Median of positional dilution of precision |
| hdopMedi | | Median of horizontal dilution of precision |
| vdopMedi | | Median of vertical dilution of precision |
| nsatusedMedi | | Median of number of satellites used |
| nsatviewMedi | | Median of number of satellites in view |
| heightMMini | | Minimum elevation |
| speedKmhMini | | Minimum speed |
| pdopMini | | Minimum positional dilution of precision |
| hdopMini | | Minimum horizontal dilution of precision |
| vdopMini | | Minimum vertical dilution of precision |
| nsatusedMini | | Minimum number of satellites used |
| nsatviewMini | | Minimum number of satellites in view |
| heightMMaxi | | Maximum elevation |
| speedKmhMaxi | | Maximum speed |
| pdopMaxi | | Maximum positional dilution of precision |
| hdopMaxi | | Maximum horizontal dilution of precision |
| vdopMaxi | | Maximum vertical dilution of precision |
| nsatusedMaxi | | Maximum number of satellites used |
| nsatviewMaxi | | Maximum number of satellites in view |
| heightMDec1 | | 10th percentile elevation |
| speedKmhDec1 | | 10th percentile speed |
| pdopDec1 | | 10th percentile positional dilution of precision |
| hdopDec1 | | 10th percentile horizontal dilution of precision |
| vdopDec1 | | 10th percentile vertical dilution of precision |
| nsatusedDec1 | | 10th percentile number of satellites used |
| nsatviewDec1 | | 10th percentile number of satellites in view |
| heightMDec9 | | 90th percentile elevation |
| speedKmhDec9 | | 90th percentile speed |
| pdopDec9 | | 90th percentile positional dilution of precision |
| hdopDec9 | | 90th percentile horizontal dilution of precision |
| vdopDec9 | | 90th percentile vertical dilution of precision |
| nsatusedDec9 | | 90th percentile number of satellites used |
| nsatviewDec9 | | 90th percentile number of satellites in view |
| nobsValid | | Number of valid GPS points |
| nobsGPS | | Number of GPS points |
| **Heart rate variables** | | |
| SDNN | | Standard deviation of RR intervals series |
| pNN50 | | Proportion of adjacent RR intervals differing by more than 50 ms |
| SDSD | | Standard deviation of differences between adjacent RR intervals |
| rMSSD | | Square root of the mean of the squares of differences between adjacent RR intervals |
| IRRR | | Difference between third and first quartile of the RR intervals series |
| MADRR | | Median of the absolute differences between adjacent RR intervals |
| TINN | | Baseline width of the triangular interpolation of the intervals histogram |
| HRVi | | Integral of the intervals histogram divided buy its maximum |
| HR | | Heart rate |
| md.prct | | Percentage of missing data |
| n.beats.bf.filter | | Number of beats before applying the filter |
| time.prct | Percentage of time for which the data is available | |
